# Modulation of hippocampal neuronal activity by *So-ochim-tang-gamibang* in mice subjected to chronic restraint stress

**DOI:** 10.1186/s12906-017-1963-1

**Published:** 2017-09-09

**Authors:** Hwa Chul Shin, Jae Ho Lee, Ki Joong Kim, Hyun Jin Shin, Jeong June Choi, Chan Yong Lee, Uk Namgung, In Chul Jung

**Affiliations:** 1 Department of Oriental Medicine, Daejeon University, Daejeon, 34520 Republic of Korea; 2 Department of Microbiology and Biotechnology, Daejeon University, Daejeon, 34520 Republic of Korea; 3 Department of Neuropsychiatry, Dunsan Korean Medicine Hospital of Daejeon University, Daejeon, 35235 Republic of Korea

**Keywords:** So-ochim-tang-gamibang, Serotonin receptor, Hippocampus, Chronic restraint stress, Depression

## Abstract

**Background:**

*So-ochim-tang-gamibang* (SOCG) is a decoction formula which has been used to improve mental activity in traditional Korean medicine. The present study was performed to evaluate whether the treatment of SOCG was involved in activating hippocampal neurons in mice which were subjected to chronic restraint stress (CRS).

**Methods:**

Mice were subjected to CRS for 2 weeks to induce depressive-like behaviors. SOCG was orally administered for the same period. mRNA expression in the hippocampus was analyzed by RT-PCR. Levels of serotonin receptor 5-HT1AR in the hippocampus were determined by western blotting and by immunofluorescence staining in coronal brain sections. Cultured neurons were prepared from the dorsal root ganglia (DRG) in mice to examine the effects of CRS and SOCG treatment on neurite outgrowth. Depressive-like behaviors of experimental animals were measured by open field test (OFT) and forced swimming test (FST).

**Results:**

mRNA levels of serotonin 1A and 1B receptors (5-HT1AR and 5-HT1BR) were decreased in the hippocampus of CRS animals and increased by SOCG treatment. Signals of 5-HT1AR protein in CA3 pyramidal cells were decreased by CRS but elevated back to levels in control animals after SOCG treatment. Phospho-Erk1/2 protein in CA3 cells showed similar pattern of changes as in 5-HT1AR, suggesting coordinated regulation after SOCG treatment in CRS animals. Axonal growth-associated protein GAP-43 levels were also decreased by CRS and then increased by SOCG treatment. In vivo administration of SOCG improved neurite outgrowth of primary DRG neurons from CRS animals and also increased 5-HT1AR protein signals. Behavioral tests of open field and forced swimming showed that immobility time periods were significantly decreased by SOCG treatment.

**Conclusions:**

Our data suggest that SOCG treatment may increase synaptic responsiveness to serotonergic neuronal inputs by upregulating 5-HT1AR in the hippocampal neurons.

## Background

Depression is a mental illness that causes a serious disability of the quality of life and affects about 20% of the population worldwide. Because of its subjectivity and qualitative nature, there has been a limitation to characterize the neurobiological basis on depression. However, recent advances in brain imaging techniques have identified the anterior cingulate cortex, hippocampus, and amygdala as susceptible brain areas to depressive illness in human [1–3]. Studies using experimental animals indicate that the intense, chronic stress activates the hypothalamus-pituitary-adrenal gland (HPA) axis, and consequently elevated glucocorticoid hormone levels cause neuronal atrophy in several brain areas.

Hippocampal granule cells and pyramidal cells establish a trisynaptic circuit which is primarily glutaminergic. Major inputs to hippocampus are given from the entorhinal cortex through the perforant path, and the findings of the long-term potentiation in hippocampal neurons after high frequency stimulation of the perforant path have paved a way to explore the physiological basis of learning and memory [4]. Hippocampus has not only a bilateral connection to amygdala, but also relays the output to several brain areas including the mammillary body and the septum through the fornix, and receives the synaptic inputs through the cingulate cortex. Thus, hippocampal activity is affected by stressful state, and, particularly in the state of stress-induced depression, the responsiveness of hippocampal neurons can be altered to serotonergic and adrenergic inputs as well as corticotropin releasing hormone (CRH) stimulation. Here, blockers or modulators of receptors against serotonin, norepinephrine and CRH have been the primary therapeutic targets for the development of antidepressants [5–7].

In traditional Korean medicine, depression is described as a congested state of *qi* and thus, the therapeutic approach for depression is to refurbish its flow by using herbal drugs and acupuncture. *So-o-chim-tang-gami-bang* (SOCG) is a modified herbal formulation of *So-o-chim-tang* by substituting *Aquilaria agallocha Roxb* with *Aucklandia lappa* and supplementing *Citrus aurantum* and *Platycodon grandifloras* [8]. It is described in *Dongui Bogam*, a classical Korean medicinal book, that *So-o-chim-tang* is effective in treating abnormal regulation of *qi* leading to pain of internal organ, and *Citrus aurantum* and *Platycodon grandiflorus* reinforce the flow of *qi* to head and neck. We have shown previously that SOCG treatment in cultured mast cells decreased the expressions of 5-hydroxytryptamine (5-HT) transporter and tryptophan hydroxylase 1 mRNAs and increased free radical-scavenging activity [9]. We further demonstrated that SOCG treatment in CRS animal model reduced corticosterone levels in the serum and induced the improvement from immobility behaviors [8]. In addition, it was reported that specific chemical ingredients or herbal components of SOCG were effective in regulating depressive- or anxiety-like behaviors in experimental animals [10–12].

While these studies strongly suggest that SOCG may play a role in regulating depressive-like behavior, studies on the role of SOCG in specific brain areas have not been reported. Based on the hypothesis that, in the nervous system, the facilitated *qi* flow would be positively linked to the increased signaling through the neural circuit, the effects of SOCG on the regulation of the *qi* flow and depression may be explored in terms of SOCG-mediated neuronal activation in brain tissues. Here, we investigated, by using CRS animal model, the effects of SOCG on hippocampal neuronal responses. Our data suggest that the activation of 5-HT1AR is involved in mediating SOCG effects on hippocampal neurons in animals of depressive-like state. The effects of SOCG were also seen from 5-HT1AR-positive neurons displaying enhanced neurite outgrowth.

## Methods

### Extraction of SOCG

Herbal drug components of SOCG were obtained from Dongkyung Pharmaceutical Company (Seoul, Korea). Preparation procedure and chemical profile of SOCG have been described in our previous report [8]. Briefly, for water extraction, a total of 11 g of SOCG, which is composed of 4 g of *Cyperi Rhizoma (Cyperus rotundus L.)*, 2 g each of *Linderae Radix (Lindera aggregata (Sims) Kosterm.), Platycodi Radix (Platycodon grandiflorum (Jacq.) A. DC.)*, and *Aurantii Fructus (Citrus aurantium L.)*, and 0.5 g each of *Aucklandiae Radix (Aucklandia lappa DC.)* and *Glycyrrhizae Radix (Glycyrrhiza uralensis Fisch.)* was boiled for 2 h, filtered, and concentrated under a reduced pressure by using the rotary vacuum evaporator. After freezing-frying, 1.6 g of the SOCG powder was obtained from 11 g of initial raw materials (14.6% of extraction ratio). SOCG powder was dissolved in purified water, filtered by using 0.22 μm Whatman filter paper (Cole-Parmer, Vernon Hills, USA) and stored at -20 °C as a stock solution (10 mg/ml) which was further diluted with saline solution (0.9% NaCl in water) for oral administration. Voucher specimens (No. 194A079–85) of collected herb samples were deposited in the herbarium of Han Kook Shin Yak Co., Ltd. (Nonsan, Korea).

### Experimental animals

C57/BL6 mice (male, 18–22 g, Samtako, Seoul, Korea) were maintained in an animal room with regulated temperature (22 °C), 60% humidity, and a 12-h light/dark cycle (light on 7 am to 7 pm). Before the experiments, animals were acclimatized for 7 days in an animal room and were allowed to eat commercial pellet chow (Samyang Co., Seoul, Korea) and drink water ad libitum. Animals were randomly divided into four groups: an intact animal control group (CTL), a chronic restraint stress with saline injection as a SOCG vehicle (CRS), and CRS plus 100 mg/kg and 300 mg/kg of SOCG-treated groups (CRS + SOCG100 and CRS + SOCG300). To induce a depressive-like state, individual animals were subject to CRS by placing in a well-ventilated 50 ml conical tube for 6 h each day for 14 consecutive days. SOCG (100 and 300 mg/kg) or an equivalent volume of saline was orally administered using a 22-gauge oral needle 2 h before CRS on a daily basis for a 2 week period. A recommended daily dose of SOCG for human is 3.2 g (Han Kook Shin Yak Co., Ltd.), which is then calculated 658 mg/kg for the use of experimental animals according to a procedure of human equivalent dose calculation (Guideline by the Food and Drug Administration, USA). However, to determine drug efficacy, we adopted a range of lower dose as much as 50% or less for a current investigation. Our recent study also indicates that SOCG doses at 100 mg/kg and 300 mg/kg are optimal for the induction of depressive-like behavior [8]. All procedures were in strict accordance with the NIH guide for the care and use of laboratory animals and approved by the Committee on Use of Live Animals for Teaching and Research at Daejeon University (Protocol number: DJUARB2014–036, Daejeon, Korea).

### Reverse transcription-polymerase chain reaction (RT-PCR)

For RT-PCR, a total 16 animals were used and equal number of animals assigned into 4 experimental groups. Hippocampus was isolated and immediately used to extract total RNA by using Easy-BLUE reagent (Intron, Sungnam, Korea). A reaction for cDNA synthesis was carried out in 30 μl using total RNA (1 μg) as a template, 1X reaction buffer (50 mM Tris-HCl, 75 mM KCl, 3 mM MgCl_2_, 10 mM DTT, 104 μM dNTP), RNasin (30 U), random primer (16 μM Promega, Madison, USA), and MMLV reverse transcriptase (200 U, Promega) for 2 h at 37 °C. For RT-PCR analysis, 30 cycles of amplification were optimal for a quantitative comparison of the target mRNA expression. The primer sequences used for PCR were the forward primers (5′-ACTCGACTTTCGGCGCTTT-3′, 5′-GCTTTGTGAACACCGACCAC-3′, 5′-GGTGTGCCTTTCCCCATCATT-3′, 5′-GGCAAGGAAGCTGGTGGTGATTT-3′, 5′-AAGAGCAGTGGAAGGACAGC-3′) and the reverse primers (5′-CTGCAAAAAGCACTGTCCCC-3′, 5′-GAGCCCGGGAGTTAATGGAG-3′, 5′-CAACATGTAGGTGATGCCCAG-3′, 5′-GGCGTGGTGGTCCTGCCAGGG-3′, 5′-TGGTATCGCCTTTGCCCATT-3′) for 5-HT1AR, 5-HT1B receptor, corticotropin releasing hormone receptor type 1 and type 2, also known as corticotropin releasing factor 1 and 2 (CRF1 and CRF2) respectively, and glucocorticoid receptor, and the forward and reverse primers (5′-TACGGATGTCAACGTCACAC-3′, 5′-CACACTGTCCCCATCTATGA-3′) for actin. PCR-amplified DNA was analyzed on agarose gels, and the band intensities were quantified using i-Solution software (Image & Microscope Technology, Burnaby, Canada).

### Immunohistochemistry

A total of 8 animals, each group consisting of 2 animals, were used for immunohistochemistry. Animals were deeply anesthetized with ketamine and xylazine, and transcardially perfused with 4% paraformaldehyde in phosphate-buffered saline (PBS). Brain was removed and postfixed for 1 h and kept overnight in 15% sucrose in PBS. Coronal brain sections of 20 μm were collected on positively charged slides and were kept at -70 °C until use. For immunofluorescence staining, tissues on the slides were fixed with 4% paraformaldehyde, 4% sucrose in PBS at room temperature for 40 min, permeablized with 0.5% nonidet P-40 in PBS, and blocked with 2.5% horse serum and 2.5% bovine serum albumin for 4 h at room temperature. Tissues were incubated with primary antibody, washed with PBST (PBS plus 0.1% triton X-100) three times for 10 min each, and incubated with fluorescein-goat anti-mouse (1:400, Molecular Probes, cat no. f2761) or rhodamine-goat anti-rabbit secondary antibodies (1:400, Molecular Probes, cat no. r6394) in 2.5% horse serum and 2.5% bovine serum albumin for 1 h at room temperature and cover-slipped with gelatin mount medium. For some experimental purpose, Hoechst staining reaction for nuclear visualization was performed between washing steps after secondary antibody reaction. Tissue sections were treated with 25 μg/ml of Hoechst 33,258 in 0.1% PBST for 10 min. The secondary antibody reaction was performed in a dark place. Images from immunofluorescence staining were captured and transferred to the computer software (ACT-1). The merged images were produced by layer blending mode options of the Adobe Photoshop (version 7.0). The primary antibodies used were anti-5-HT1AR (1:300, Abcam, cat no. ab85615), anti-phospho-Erk1/2 kinase (1:800, Sigma, cat no. 9101 L), anti-GAP-43 (1:800, Santa Cruz Biotech, cat no. ab16054) and anti-NF-200 (1:400, Sigma, cat no. N0142) antibodies.

### Western blot analysis

A total of 16 animals, each group consisting of 4 animals, was used to dissect hippocampal tissues. Isolated hippocampi were washed with ice-cold PBS, and sonicated under 50–200 μl of triton lysis buffer (20 mM Tris, pH 7.4, 137 mM NaCl, 25 mM β-glycerophosphate, pH 7.14, 2 mM sodium pyrophosphate, 2 mM EDTA, 1 mM Na_3_VO_4_, 1% triton X-100, 10% glycerol, 5 μg/ml leupeptin, 5 μg/ml aprotinin, 3 μM benzamidine, 0.5 mM DTT, 1 mM PMSF). Protein (15 μg) was resolved in 12% SDS polyacrylamide gel and transferred to immobilon polyvinylidenedifluoride (PVDF) membranes (Millipore, Bedford, USA). Blots were blocked with 5% nonfat dry milk in PBST (17 mM KH_2_PO_4_, 50 mM Na_2_HPO_4_, 1.5 mM NaCl, pH 7.4, and 0.05% Tween-20) for 1 h at room temperature and then incubated overnight at 4 °C in 0.1% triton X-100 in PBS plus 5% nonfat dry milk containing primary antibodies. Protein bands were detected by using the Amersham ECL kit (Amersham Pharmacia Biotech, Piscataway, USA), with horse radishperoxidase-conjugated goat anti-rabbit (1:400, Life Technologies, cat no. R6394) or goat anti-mouse secondary antibodies (1:400, Life Technologies, cat no. F2761). Transduction Laboratories, Lexington, USA). Primary antibodies used in the present study were anti-phospho-Erk1/2 kinase (1:4000, Cell Signaling, cat no. 9101 L), anti-GAP-43 (1:4000, Cell Signaling, cat no. ab16054), and anti-actin (1:10,000, MP Bio, cat no. A1978) antibodies.

### DRG neuron culture and analysis of neurite outgrowth

Mice (C57BL/6) were subjected to a 2 week period of CRS and SOCG administration as described above, and 24 h after the last constraint, DRG at lumbar 4–5 levels were dissected for primary neuron culture as described previously [13]. A total of 16 animals, each group consisting of 4 animals, was used to prepare DRG neurons. DRG isolated from each experimental group were pooled to prepare neuron culture. We used DRG neurons for in vitro SOCG study because primary DRG neurons, unlike brain neurons including hippocampal neurons, can be prepared from adult animals and maintained in a stable condition for several days. Here we assumed that the extent of neurite outgrowth might reflect in vivo neural activity including depression-like brain activity. For the preparation of DRG neurons from mice given sciatic nerve injury (SNI), sciatic nerves on the left side were exposed on the middle thigh and a crush injury was given by holding a nerve with the forceps for 30 s twice as described previously [14], and the DRG at left lumbar 4–5 were prepared at 7 d post injury. A total of 8 animals, in which 4 animals were subjected to SNI and another 4 animals were non-surgery control (CTL), were used to prepare DRG. Dissociated cells were plated onto 12 mm coverslips (Marlenfeld GmbH & Co. KG, Lauda-Königshofen, Germany) precoated with poly-L-ornithine (0.1 mg/ml; Sigma) and laminin (0.02 mg/ml, BD Bioscience, San Diego, USA) and cultured for 24 h before the harvest. Immunofluorescence staining of cells was essentially same as those of brain sections on the slides. Antibodies used were anti-neurofilament-200 (NF-200) (1:400, Sigma, cat no. N0142) and anti-5-HT1AR (1:300, Abcam, cat no. ab85615) antibodies. Digital images of neuronal process were captured and transferred to the Adobe Photoshop (version 7.0). The length of neurite processes exhibiting clear outgrowth (longer than cell diameter) from the cell body was analyzed by using i-Solution software program (Image and Microscope Technology).

### Behavioral tests

Animals were placed in a room for behavioral tests and subjected to preliminary swimming for 10 min. Three hours later, FST was performed in a transparent, cylindrical plexglas container (20 cm diameter, 46 cm height) filled with tap water (20 cm height, 20 °C). Animal movement was monitored for 6 min by video tracking software (SMART 3.0; Panlab, Barcelona, Spain) and the immobility time was measured for the last 4 min. For OFT, animals were placed in a room and adjusted overnight before the test. Animal was placed in a white-colored plexglas box (30 × 30 × 30 cm^3^) and its movement was monitored for 10 min following an initial 1 min of adjustment period. Immobility time was measured by using video tracking software (SMART 3.0; Panlab S.L.U., Barcelona, Spain). For both tests, 4 animals per experimental group were used and average immobility time was compared among experimental groups.

### Statistical analysis

Data were presented as mean ± standard error of mean (SEM). The mean number data in individual groups were compared by one-way ANOVA followed by Tukey test (SPSS computer software version 12.0), and statistically significant differences were reported as *p < 0.05, **p < 0.01, ***p < 0.001.

## Results

**Fig. 1:**
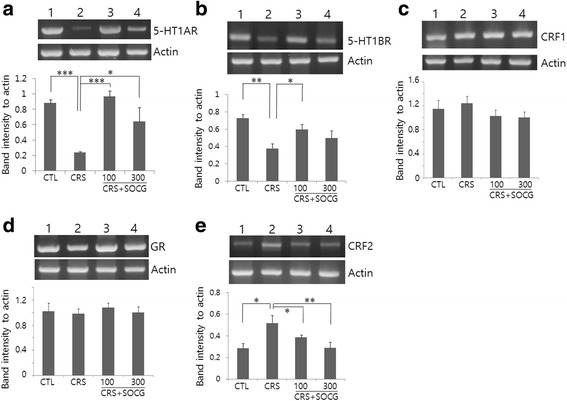
RT-PCR analysis for the expression of stress-related genes in the hippocampus. Hippocampal mRNA from different animal groups was used for RT-PCR using primers of 5-HT1AR (**a**), 5-HT1BR (**b**), CRF1 (**c**), glucocorticoid receptor (GR; **d**), and CRF2 (**e**). RT-PCR for actin mRNA was performed as a loading control. For all figures **a** through **e**, lane 1, CTL; lane 2, CRS; lane 3, CRS + 100 mg/kg SOCG; lane 4, CRS + 300 mg/kg SOCG. The ratio of band intensity of each mRNA to actin was shown by bar graphs (*n* = 4). *P < 0.05, **P < 0.01, ***P < 0.001 (One-way ANOVA, n = 4)

To examine whether SOCG treatment in CRS mice alters the expression of stress-related genes in the hippocampus, expressions of mRNAs encoding 5-HT1AR, 5-HT1BR, CRF1 and 2, and glucocorticoid receptor were analyzed by RT-PCR. 5-HT1AR mRNA was expressed in control animals and was significantly decreased after CRS (Fig. 1a). 5-HT1BR mRNA levels were similarly decreased by CRS and then elevated by SOCG treatment. SOCG at 100 mg/kg upregulated 5-HT1BR mRNA levels similar to those in control animals. The extent of mRNA increase at a higher dose (300 mg/kg) was less effective than a lower dose (Fig. 1b). CRF1 and GR mRNA levels remained constant by CRS and SOCG (Fig. 1c,d). In case of CRF2, mRNA levels were significantly increased by CRS but decreased to normal levels after SOCG treatment (Fig. 1e).

**Fig. 2:**
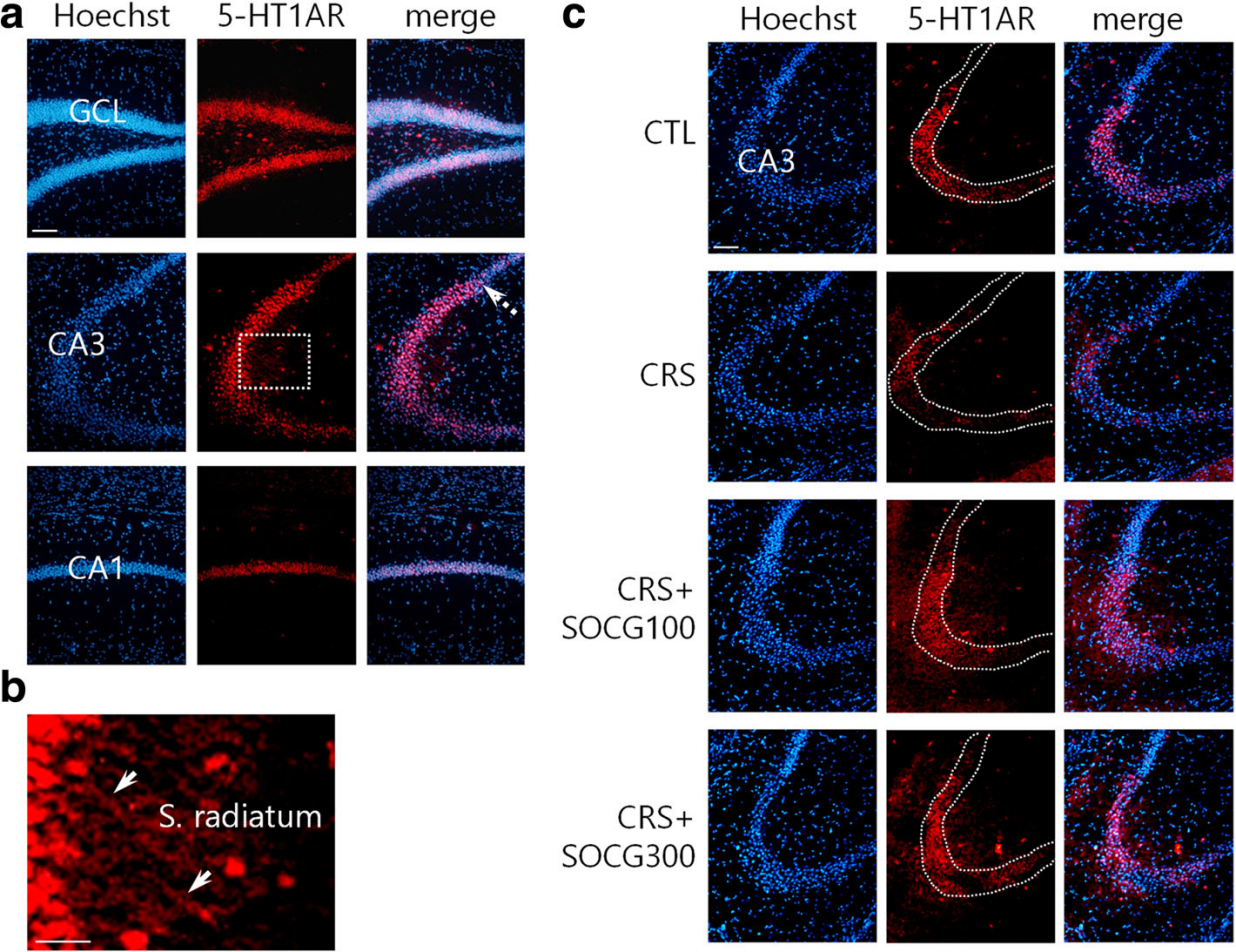
Immunohistochemical analysis of 5-HT1AR protein signals in the hippocampal subfields. **a** 5-HT1AR signals in the granule cell layer (GCL), CA3, and CA1 areas. Notice the remarkable decreases of signals at the boundary to CA2 area (marked dotted arrow). **b** 5-HT1AR signals in the striatum radiatum (Enlarged view for a rectangular area in **a**). In (**a**) and (**b**), coronal brain sections from CTL animals were used for immunostaining analysis. **c** 5-HT1AR signals in the CA3 subfields for different animal groups. Scale bars in (**a**) and (**c**): 200 μm, bar in (**b**): 100 μm

RT-PCR analysis indicated that 5-HT1AR mRNA expression was regulated most efficiently among several mRNAs examined in the hippocampus by SOCG treatment. To determine 5-HT1AR protein signals in the hippocampal subfields, coronal brain sections were used for immunohistochemical analysis. In control animals, 5-HT1AR signals were observed in major hippocampal cell body layers including dentate gyrus granule cell and CA3 and CA1pyramidal cell layers (Fig. 2a). Protein signals were seen additionally in the striatum radiatum of CA3 region (arrows in Fig. 2b). 5-HT1AR signals in CA3 pyramidal cells were decreased after CRS to a large extent, but were increased by SOCG treatment (Fig. 2c). In the CA1 and granule cell layers, moderate levels of protein signals were observed similarly among experimental groups (data not shown).

**Fig. 3:**
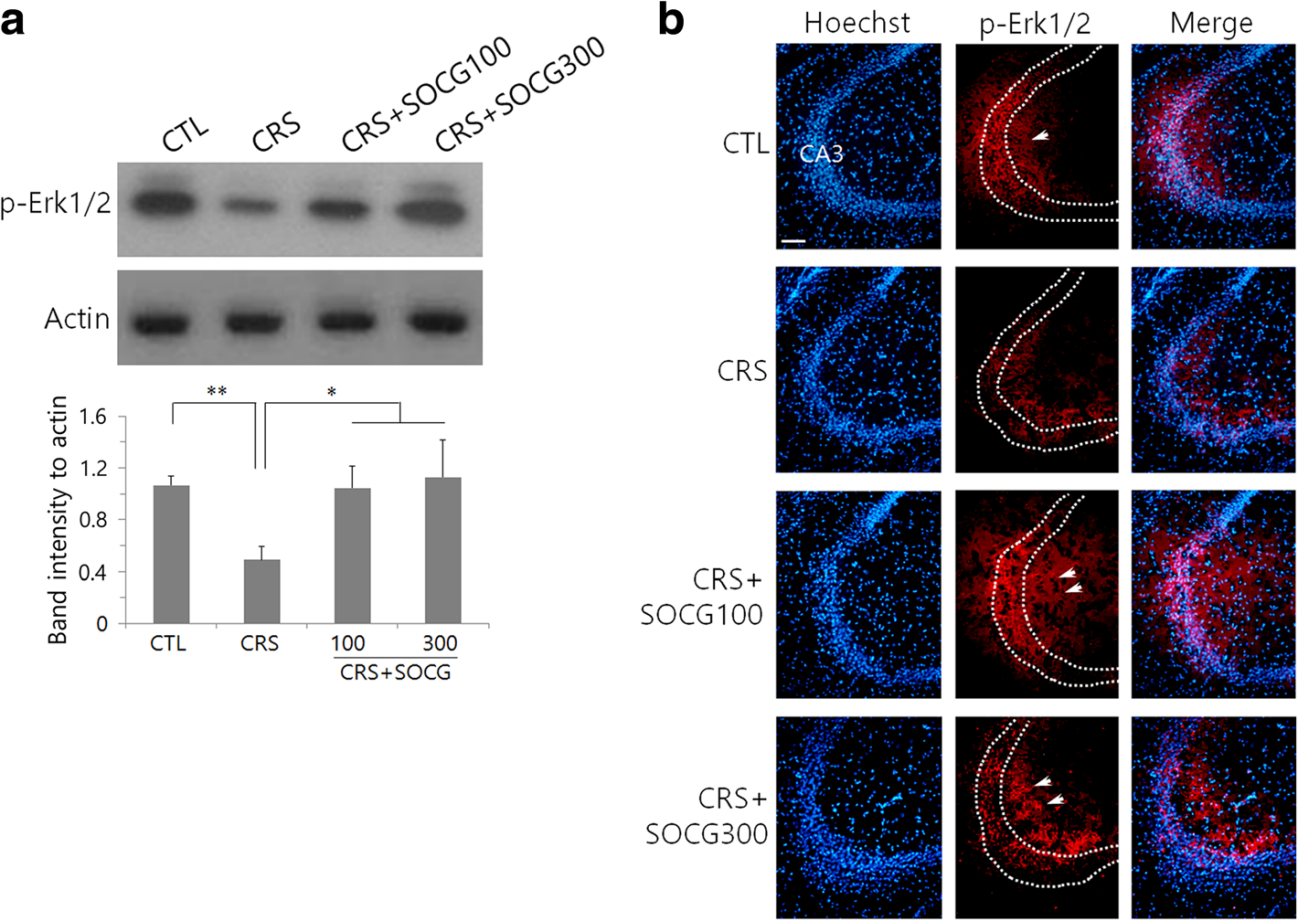
Changes of phospho-Erk1/2 by SOCG treatment in the hippocampus of CRS animals. **a** Western blot analysis of phospho-Erk1/2 in the hippocampus. Images in the upper panel show the representatives from 4 independent experiments, and quantitation of protein band intensity relative to actin control are shown in the lower panel. **P < 0.01, **P < 0.01 (One-way ANOVA, n = 4). **b** Subfield distribution of phospho-Erk1/2 signals in the hippocampal CA3 area. Scale bar in (B): 200 μm

To examine hippocampal neuronal activation after CRS and SOCG treatment, we analyzed phospho-Erk1/2 protein signals. Phospho-Erk1/2 levels were significantly decreased after CRS but then increased by SOCG treatment (Fig. 3a). Similar pattern of phospho-Erk1/2 signals was observed in CA3 neurons which showed a decrease after CRS and an increase by SOCG treatment (Fig. 3b). In addition to CA3 pyramidal cell layer, extra-phospho-Erk1/2 signals were observed in the striatum oriens and striatum radiatum areas in SOCG-treated CRS animals as well as in controls (arrows in Fig. 3b). Protein signals in other hippocampal areas were relatively weaker than CA3 region, and any remarkable changes were not observed by CRS and SOCG treatment (data not shown).

**Fig. 4:**
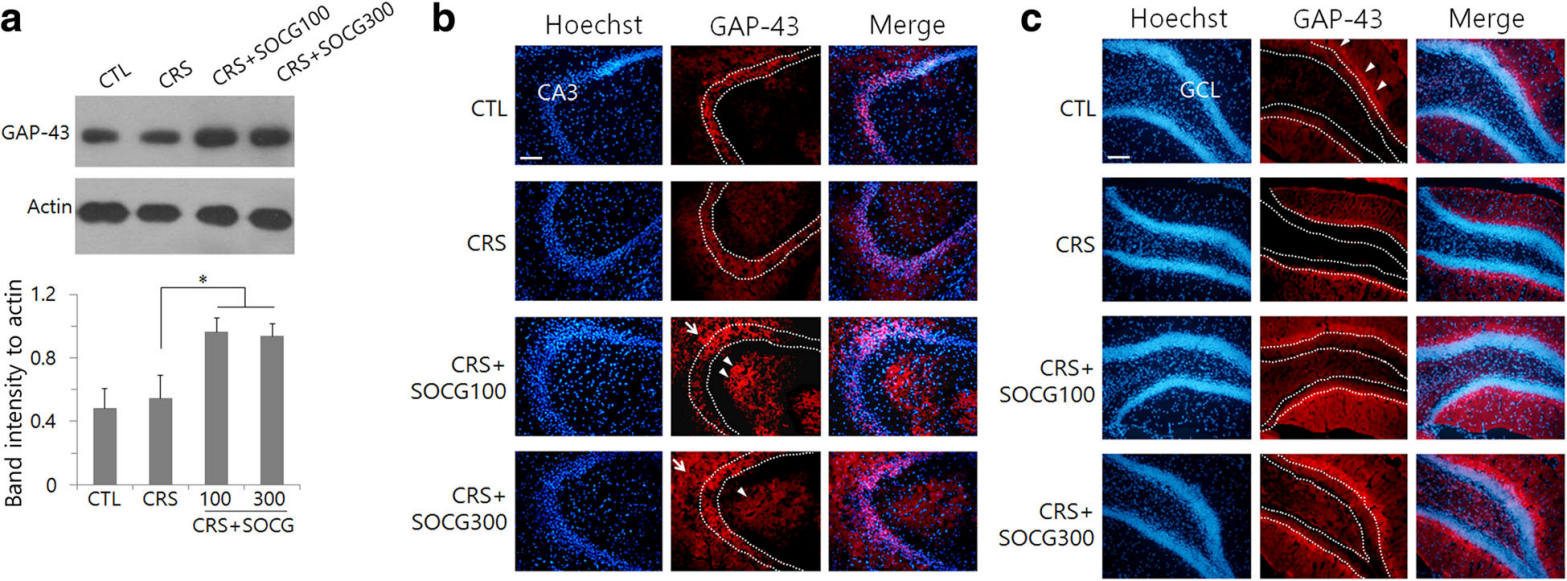
Regulation of GAP-43 levels in the hippocampus by SOCG treatment in CRS animals. **a** Western blot analysis of GAP-43 in the hippocampal tissue. Images in the upper panel show the representatives from 4 independent experiments, and quantitation of protein band intensity relative to actin control are shown in the lower panel. *P < 0.05. (One-way ANOVA, n = 4). **b** Immunohistochemical analysis of GAP-43 in the CA3 region. GAP-43 signals were seen in the striatum oriens (arrows) and striatum radiatum (arrowheads) besides CA3 pyramidal cell layer. **c** GAP-43 signals in the dentate gyrus. GAP-43 signals were observed clearly in the inner molecular layer (arrowheads), but rarely seen from the granule cell layer (GCL). Scale bars in (**b**) and (**c**): 200 μm

In adult rodents, constitutive expression of GAP-43 occurs in the CA3 and CA1 pyramidal neurons, but not in the granule cells [15]. Western blot analysis for hippocampal proteins showed that GAP-43 level was not changed by CRS but were increased by SOCG treatment (Fig. 4a). In CA3 hippocampal subfield, GAP-43 signals were observed similarly in animal groups treated with CRS and SOCG, but additional signals were seen in the striatum oriens (arrows) and striatum radiatum (arrowheads) in SOCG-treated animals (Fig. 4b). In the dentate gyrus, GAP-43 signals were observed in the inner molecular layer, but no signals were found in the granule cell layer (arrows in Fig. 4c). GAP-43 signals in the inner molecular layer of the dentate gyrus were lower in the CRS animals compared to the controls, but were increased by SOCG treatment.

**Fig. 5:**
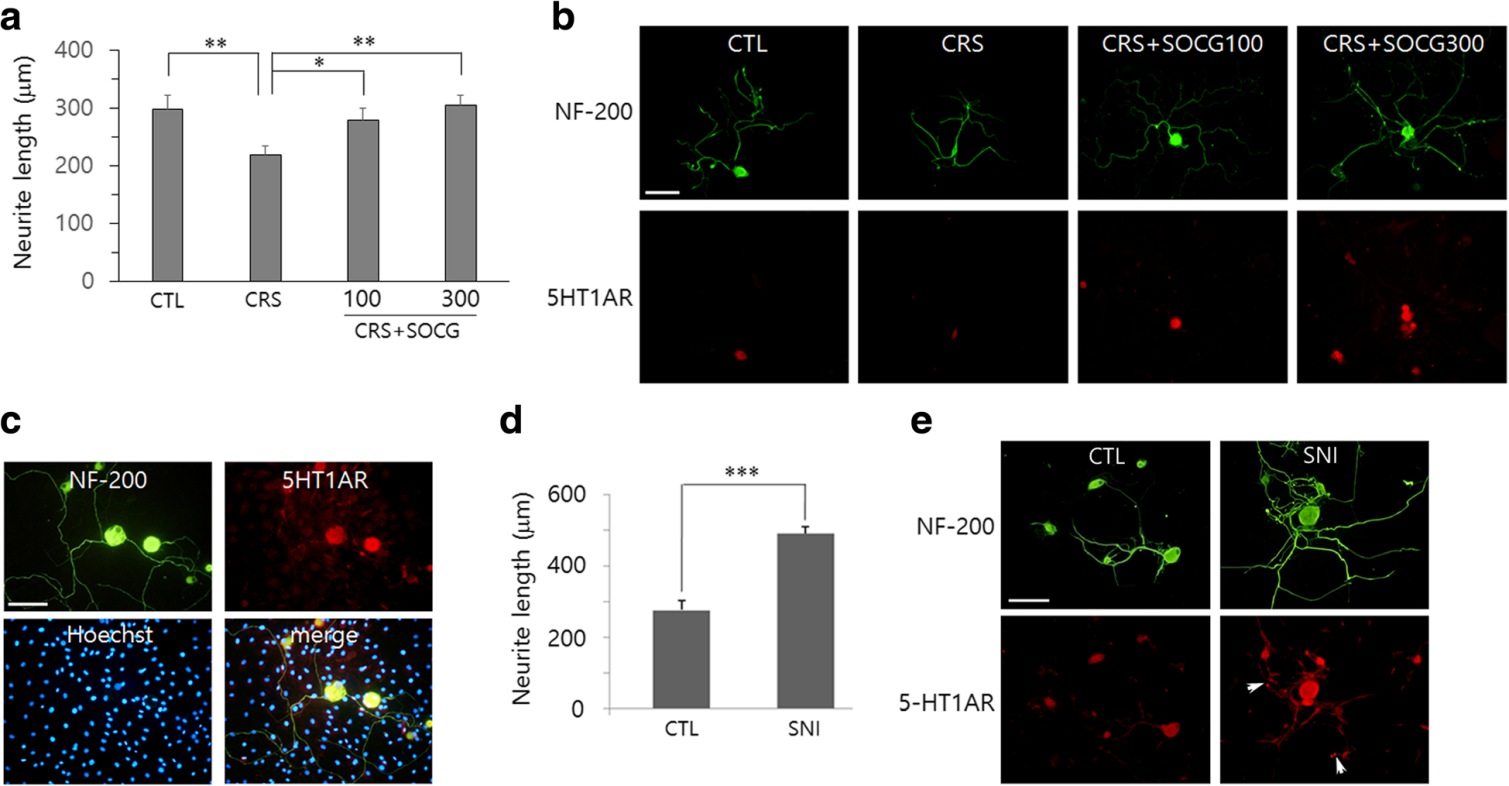
Effects of SOCG treatment on the neurite outgrowth of DRG neurons. **a**-**c**. DRG at lumbar 4 and 5 were prepared from animals from 4 experimental groups. Neuritic processes of cultured DRG neurons were visualized by NF-200 staining (in green) and quantified (**a**). Representative images of DRG neurons for 4 experimental groups (**b**), and those from CRS + SOCG300 group (**c**). **d**-**e**. DRG neurons, which were prepared from the animal given sciatic nerve preinjury (SNI), were used for immunofluorescence staining with anti-NF-200 and anti-5-HT1AR antibodies. *P < 0.05; **P < 0.01 (One-way ANOVA, n = 4). All scale bars in (**b**), (**c**) and (**e**): 100 μm

We further investigated whether CRS and SOCG affected the neurite outgrowth of cultured neurons. As shown in 5A and B, the neurite length was significantly decreased in CRS animals compared to CTL animals, and then increased by SOCG treatment. 5-HT1AR signals were very weak in neurons from CRS animals, but its intensity was increased by SOCG treatment (Fig. 5b). In CRS + SOCG animal, 5-HT1AR signals were detected in the soma and neuritic processes in DRG neurons, but were not found in non-neuronal cells (e.g., Schwann cells) that co-existed in culture and were identified by Hoechst nuclear staining (Fig. 5c). Enhanced neurite outgrowth was clearly seen in DRG neurons which had been prepared from SNI animals (Fig. 5d). 5-HT1AR signals were also increased after SNI and showed its distribution in the neuritic processes as well as in the soma (arrows in Fig. 5e).

**Fig. 6:**
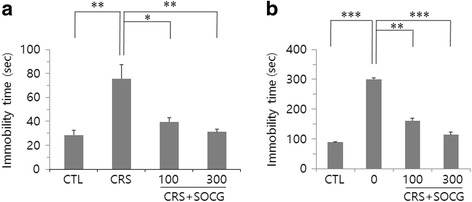
Regulation of depressive-like behavior by SOCG administration in CRS animals. Animals in each experimental groups were subjected to FST (**a**) and subsequently by OFT (**b**) 24 h later. *P < 0.05, **P < 0.01, ***P < 0.001 (One-way ANOVA, n = 4)

To examined whether CRS and SOCG treatment influenced on depressive-like behaviors, we performed FTS and OFT. In FST, animals in CRS group showed a significant increase in the duration of immobility compared to control animals. Then, immobility time was decreased by SOCG administration (Fig. 6a). We further found that immobility time in OFT was significantly increased after 2 week CRS, but decreased by SOCG treatment (Fig. 6b). Regulation of depressive-like behaviors in both FST and OFT were more effective with 300 mg/kg of SOCG than 100 mg/kg.

## Discussion and conclusion

SOCG is a modified prescription of *So-o-chim-tang*, and has been used in Korean traditional medicine for the treatment of congested vital energy. According to traditional medicinal theory, a prolonged stagnation of *qi* and blood flow and unbalance of yin-yang cause the symptom of depression. Thus, SOCG or some modified decoctions of SOCG have been prescribed in traditional medicinal therapy. However, the biological basis on its efficacy is largely unknown, and only recently has the potential effect on depressive-like disorders begun to be investigated [8]. Here, we extended the effects of SOCG on the regulation of molecular factors that may be involved in neuronal activities in the hippocampus of CRS animals.

Hippocampus has neural inputs from the entorhinal cortex, prefrontal cortex, brainstem reticular formation, and others, and sends out efferents to mammillary body through fornix, entorhinal cortex, amygdala, and cingulate gyrus. Brain imaging studies indicate a reduction of hippocampal volume in patients with manic depressive disorder [16, 17], and hippocampal volume was reported to increase in patient after 3 years of antidepressant therapy [18]. Extensive, chronic stress can cause depressive disorder. Here, stress abnormally regulates the HPA axis, and leads to increased release of glucocorticoid, which in turn may affect adversely on the survival of hippocampal neurons [19]. Besides glucocorticoid, hippocampus receives serotonergic and adrenergic inputs mainly from the brainstem reticular formation, and their potential role for the regulation of anxiety and depression has been well documented [20–22].

CRS elevates serum corticosterone through the activation of HPA axis. During chronic stress state, CRH mediates neuronal activation by acting on their receptors in addition to stimulation of ACTH secretion in the pituitary gland. Two main types of CRH receptors, CRF1 and CRF2, are known to be functionally involved in the regulation of stress responses [23, 24]. CRF1 and CHF2 as well as CRH are expressed in hippocampal neurons, and are involved in activation of hippocampal neurons in response to stress. Here, prolonged, external stress signals such as glucocorticoid and neuronal inputs from the amygdala could augment hippocampal neuronal responses and may cause structural and functional alterations of hippocampal neurons [25]. It was further reported that the activation of CRF1 can mediate anxiolytic and anxiogenic properties between forebrain glutaminergic and midbrain dopaminergic neurons [26]. We found clear expression of CRF1 and glucocorticoid receptor (GR) mRNA in the hippocampus, but no changes were observed by CRS. Yet, CRF2 mRNA levels were increased by CRS and downregulated by SOCG. It was previously reported that hippocampal expression of CRF1 and CRF2 was moderate and similar among hippocampal subfields though slight variation was noted [27]. Further studies are required to define the significance of CRS-induced CRF2 in the hippocampus and the role of intervention of SOCG.

Previous studies have documented that activation of serotonergic pathway in the brain plays an important role in regulating depressive disorder, as is widely recognized by the clinical use of selective serotonin reuptake inhibitors (SSRIs) [28]. There are several types of serotonin receptors expressed in brain regions including raphe nucleus, hippocampus, cerebral cortex, septum, amygdala, and others, and activation of some serotonin receptors have antidepressant effects [29]. Notably, 5-HT1AR in the hippocampus and cortex was shown to be required for developmental regulation of anxiety-like behavior and antidepressant response [30, 31]. Studies using agonist of serotonin 1A receptor or gene knockout animals reported the involvement of hippocampal serotonergic neuronal activity in behavioral expression of anxiolytic or anxiogenic properties [32–34]. Furthermore, activation of serotonine1B receptor in the brain via the interaction with p11 was positively linked to behavioral rescue by antidepressant therapy [35]. Here our data show that, unlike CRF and glucocorticoid receptor, 5-HT1A and 1B receptors were largely decreased by CRS and increased again by SOCG. Since the changes of 5-HT1AR expression was more dramatic than 5-HT1BR, we focused our study on 5-HT1AR in the hippocampal subfields. While 5-HT1AR signals were found in major subfields, signals in the CA3 area were detected in the cell layer and striatum radiatum. Interestingly, 5-HT1AR signals were largely decreased by CRS and regained by SOCG treatment. To understand possible signaling events related to 5-HT1AR upregulation in CA3 neurons by SOCG treatment in CRS animals, we examined phopsho-Erk1/2 levels in the hippocampal neurons. It was noted that the pattern of upregulation of phospho-Erk1/2 by SOCG treatment was similar to that of hippocampal 5-HT1AR protein signals. 5-HT1AR and phospho-Erk1/2 signals in CA3 area were commonly observed in striatum radiatum where the synaptic inputs of mossy fibers are delivered. Activation of 5-HT1AR in response to serotonergic neuronal inputs can induce intracellular signaling events via the activation of phospho-Erk1/2 [36]. Thus in SOCG-treated animals, CA3 neuronal activation via 5-HT1AR signaling may contribute to the reorganization of neural circuit in the brain.

GAP-43 is a neural protein that has been expressed in many brain tissues although it is inducible in neurons undergoing development and axonal regeneration [37, 38]. Given the notion that GAP-43 expression in adult hippocampus is related to synaptic plasticity [39], it is conceivable that its expression may be downregulated in animals with depression because depression may reflect an alteration in the organization of neural circuits in the brain. While overall GAP-43 protein in the hippocampal tissue was consistent without any conspicuous change after CRS, significant upregulation was induced by SOCG treatment. In CA3 area, neuronal GAP-43 signals were detected in the inner- and outer pyramidal layers. Since GAP-43 protein is selectively transported into axons, it is likely that GAP-43 signals in this region may be localized in axons (e.g., commissural fibers) transmitting synaptic inputs to CA3 neurons and axons from hilus region, but not mossy fibers (see below). GAP-43 in the dentate gyrus showed clear signals in the outer molecular layers of the dentate gyrus, but no signals were observed in granule cells and thus no GAP-43 in its axons (mossy fiber). It is thus speculated that incoming fibers (perforant path) from entorhinal cortex in SOCG + CRS animals may have an augmented synaptic connectivity to dentate granule cells possibly by involving hilar interneurons, which in turn, relay enhanced outputs to CA3 neurons. CA3 neurons, also showing increased GAP-43 expression, could enhance synaptic plasticity in its connectivity to the targets such as CA1 neurons and contralateral neurons.

Since we found induction of GAP-43 in hippocampal neurons by SOCG, we reasoned that there may be a commonality in the responsiveness of neurons in both central and peripheral nervous systems. To this end, DRG neurons prepared from CRS and CRS + SOCG animals were analyzed. In CRS + SOCG animal group, length of neurite was significantly increased and 5-HT1AR signals were increased as well. Sciatic nerve axons can regenerate after injury and induce GAP-43 expression strongly in regenerating axons. In parallel, DRG neurons, which were prepared from animals undergone sciatic nerve injury for 3–7 days, represent enhanced neurite outgrowth and increased GAP-43 expression [40, 41], which is interpreted as transcriptional activation of regeneration-related target genes in response to retrograde lesion signals from the peripheral injury site [42]. Our data showed that DRG neurons prepared from animals given ‘preconditioned’ peripheral nerve injury showed increased expression of 5-HT1AR in addition to GAP-43, suggesting that SOCG-mediated signaling evens mediated by 5-HT1AR and GAP-43 expression may be related to growth processes of neural circuits in the hippocampus. Thus, we conclude that SOCG activates the signaling events in the hippocampal neurons of CRS animals. Consequent activation of hippocampal neuronal circuit may contribute to behavioral rescue as anxiolytic effects by activating limbic system. This notion is supported by our data demonstrating an improvement of depressive-like behavior by SOCG treatment. Further studies on SOCG may have a great potential in developing therapeutic strategies as well as for the exploration of mechanistic basis on its action.

## Abbreviations

5-HT1AR: Serotonin 1A receptor
5-HT1BR: Serotonin 1B receptors
CRF1: Corticotropin-releasing hormone receptor type 1 receptor
CRF2: Corticotropin-releasing hormone receptor type 2 receptor
CRS: Chronic restraint stress
sciatic nerve injury: SNI
SOCG: So-ochim-tang-gamibang
